# Accelerating global left-ventricular function assessment in mice using reduced slice acquisition and three-dimensional guide-point modelling

**DOI:** 10.1186/1532-429X-13-49

**Published:** 2011-09-14

**Authors:** Alistair A Young, Debra J Medway, Craig A Lygate, Stefan Neubauer, Jürgen E Schneider

**Affiliations:** 1 Department of Anatomy with Radiology, University of Auckland, Auckland, New Zealand; 2 Department of Cardiovascular Medicine, University of Oxford, UK

## Abstract

**Background:**

To investigate the utility of three-dimensional guide-point modeling (GPM) to reduce the time required for CMR evaluation of global cardiac function in mice, by reducing the number of image slices required for accurate quantification of left-ventricular (LV) mass and volumes.

**Methods:**

Five female C57Bl/6 mice 8 weeks post myocardial infarction induced by permanent occlusion of the left coronary artery, and six male control (un-operated) C57Bl/6 mice, were subject to CMR examination under isoflurane anaesthesia. Contiguous short axis (SAX) slices (1 mm thick 7-9 slices) were obtained together with two long axis (LAX) slices in two chamber and four chamber orientations. Using a mathematical model of the heart to interpolate information between the available slices, GPM LV mass and volumes were determined using full slice (all SAX and two LAX), six slice (four SAX and two LAX) and four slice (two SAX and two LAX) analysis protocols. All results were compared with standard manual volumetric analysis using all SAX slices.

**Results:**

Infarct size was 39.1 ± 5.1% of LV myocardium. No significant differences were found in left ventricular mass and volumes between the standard and GPM full and six slice protocols in infarcted mice (113 ± 10, 116 ± 11, and 117 ± 11 mg respectively for mass), or between the standard and GPM full, six and four slice protocols in control mice, (105 ± 14, 106 ± 10, 104 ± 12, and 105 ± 7 mg respectively for mass). Significant differences were found in LV mass (135 ± 18 mg) and EF using the GPM four slice protocol in infarcted mice (p < 0.05).

**Conclusion:**

GPM enables accurate analysis of LV function in mice with relatively large infarcts using a reduced six slice acquisition protocol, and in mice with normal/symmetrical left-ventricular topology using a four slice protocol.

## Background

Genetically manipulated mouse models are useful for studying the genetic determinants of cardiac disease. Surgical and pharmacological interventions are routinely performed to evaluate disease and treatment in these mouse models. Cardiovascular magnetic resonance (CMR) has been shown to provide accurate and precise non-invasive measures of cardiac function in control and chronically infarcted mice [1-4]. Typically, contiguous short axis (SAX) slices are acquired covering the left ventricle (LV). The inner and outer contours of the LV are manually segmented in frames with minimal (i.e. end-systolic) and maximal (i.e. end-diastolic) ventricular volumes, and the slices summed to give total left ventricular (LV) volume and mass. A typical study may require several loading conditions or pharmacological stress to examine LV functional performance [5]. The total imaging time required for the evaluation of LV mass and volume therefore represents a major bottleneck for high throughput evaluation of mouse models. Recently, three-dimensional (3D) guide point modeling (GPM) has been shown to achieve fast evaluation of LV mass and volumes in normal mice [6]. This method enables a 3D geometric model of the LV to be interactively customized to the geometry and motion of the particular animal. Information from both short (SAX) and long axis (LAX) slices are combined into a coherent spatio-temporal model.

Since GPM utilizes a mathematical model, which interpolates information from the available SAX and LAX slices, the need for contiguous slices may be avoided. GPM may therefore enable the total scan time to be reduced by only requiring a reduced number of slices. Since information is correlated between slices, the model may be able to interpolate information between slices without a marked reduction in accuracy. In a study in human patients with myocardial infarction, GPM was utilized with a reduced slice acquisition protocol consisting of four SAX and two LAX slices, acquired in a single breath-hold [7]. This was found to give accurate and reproducible estimates of LV mass and volume comparable with standard lengthy acquisition methods [7]. Here, we investigate the utility of GPM to reduce the number of slices, and hence the image acquisition time, required for accurate determination of LV mass and volumes in murine studies of chronic myocardial infarction. Reducing the image acquisition time would have a major beneficial effect on the health of the animal, by reducing the anesthetic burden. Also, higher throughput would be enabled for studies, which require phenotype screening. Alternatively, shorter protocols would enable additional loading conditions or pharmacological stress conditions to be evaluated in a reasonable time frame.

This study sought firstly to evaluate the accuracy of the 3D GPM analysis method for the estimation of cardiac function in mice with myocardial infarction. Secondly, we investigated the impact of reducing the number of acquired slices on the accuracy of cardiac functional parameters in control and in chronically infarcted mice. Global cardiac functional parameters, including end-diastolic volume (EDV), end-systolic volume (ESV), stroke volume (SV), ejection fraction (EF), and LV mass (LVM) were assessed in both control mice and mice with myocardial infarction using a 3D GPM analysis of i) all slices, ii) a six slice protocol including four SAX and two LAX slices, and iii) a four slice protocol including only two SAX and 2 LAX slices. Each method was compared against the standard evaluation procedure of manual segmentation and slice summation of contiguously acquired slices. We hypothesized that i) 3D GPM can achieve results of similar accuracy to the standard analysis in mice with myocardial infarction using all slices; ii) the six slice protocol can be employed with GPA in mice with or without myocardial infarction; and iii) the four slice protocol can be used in situations without regional disease/remodelling in which a fast estimate of ventricular function is required within a very short time frame.

## Methods

### Animal Preparation and Infarct Model

C57BL/6 mice were obtained from a commercial breeder (Harlan, UK) at least one week prior to the first imaging time point to allow naturalization to new surroundings, and were kept under controlled conditions for temperature, humidity and light, with chow and water available *ad libitum*. After induction of anesthesia in an anesthetic chamber using 4% isoflurane in 100% oxygen, the animals were positioned prone on a dedicated mouse cradle and maintained at 1.5-2% isoflurane at 2 l/min oxygen flow throughout the CMR experiments. Temperature was maintained at ~37°C using a warm air blanket. Cardiac and respiratory signals, derived from subcutaneously inserted needles and from a pressure pad, respectively, were continuously monitored using an in-house developed ECG and respiratory gating device [1].

Chronic myocardial infarction was performed by permanent ligation of the left anterior descending coronary artery as previously described [8]. In brief, adult C57Bl/6 mice were anesthetized with 2% isoflurane in 100% O_2_, intubated and ventilated with a tidal volume 250 μl and respiratory rate 150 breaths/min (Hugo-Sachs MiniVent type 845, Harvard Apparatus, UK). A left thoracotomy was performed in the fourth intercostal space. An intramyocardial suture was placed 1-2 mm below the atrio-ventricular groove with an atraumatic needle and a 6-0 polyethylene suture. Mice were given subcutaneous buprenorphine (0.8 mg/kg) for pain relief. Six control (male, 26.2 ± 1.1 g) and five mice with 8 weeks old myocardial infarction (female, 24.8 ± 2.2 g) were subjected to CMR (data from full slice analysis of the control mice have been reported previously [6]).

All procedures conformed to Home Office *Guidance on the Operation of the Animals (Scientific Procedures) Act*, 1986 (HMSO) and to institutional guidelines.

### Imaging Experiments

Imaging experiments were carried out on a 9.4T (400 MHz) MR system (Varian Inc., Palo Alto, CA) comprising a horizontal magnet (bore size 210 mm), a VNMRS Direct Drive™ console, and a shielded gradient system (1000 mT/m, rise time 130 μs). A quadrature birdcage volume coil (Rapid Biomedical, Rimpar, Germany) 33 mm in diameter was used for transmission and reception. After scouting, manual tuning and matching the coil, and slice selective shimming, ECG and respiratory gated 2D spoiled gradient echo cine images were acquired in the SAX orientation in 7-9 contiguous slices, 1 mm thick, covering the left ventricle as described previously [1]. The imaging parameters were: flip angle 15°, TE/TR = 1.8/4.6 ms; field-of-view (FOV) = 25.6 × 25.6 mm, bandwidth = 147 kHz, matrix size 256 × 256. Following the SAX slices, two LAX slices were also acquired, one in the two-chamber and one in the four-chamber view, respectively. Each slice required 2-3 mins (depending on heart- and respiratory rate) to acquire, and the entire protocol took 30-40 mins (including the setting up time which was about 10 min). Data reconstruction was performed off-line using purpose written IDL-software (ITT, Crowthorne, UK). All raw data were isotropically zerofilled by a factor of two and filtered using a modified 3rd order Butterworth filter before Fourier transformation, resulting in an in-plane reconstructed pixel size of 50 × 50 μm^2^.

### Standard Analysis

For each mouse, LVM and EDV and ESV volumes were determined by manual segmentation and slice summation of the contiguous slices, as described previously [1]. A single operator, blinded to animal ID, analyzed all SAX slices using Amira 4.1.2 (TGS/Mercury Systems, Merignac Cedex, France). The number of voxels of each compartment multiplied by the voxel size yielded the respective volumes. LV mass was obtained by multiplying the volume with the specific gravity of 1.05 g/cm^3 ^and averaging end-diastolic and end-systolic frames. The functional parameters stroke volume (SV = EDV - ESV) and ejection fraction (EF = SV/EDV) were calculated. End-diastolic and end-systolic frames were selected according to maximal and minimal ventricular volume for each slice [4]. Infarct size was determined as the relative area of akinetic myocardium as described previously [4].

### Guide Point Modeling Analysis

GPM was performed by a single operator blinded to animal ID and the standard results. A 3D mathematical model of the LV geometry was interactively customized to each case, using a similar methodology to that previously validated in humans [9] and mice [6]. Briefly, the geometry was adapted to each case by mathematical optimization to guide-points provided by the user, and image derived data points provided by an image processing algorithm. Information from both SAX and LAX slices is interpolated in 3D and across time, in order to produce a smoothly varying, coherent geometry and motion based on the available data.

LV volumes were calculated by numerical integration of the curved surfaces represented by the mathematical model, up to the mitral valve plane (defined as the 3D plane best fitting the locations of the four mitral valve points defined in the two LAX slices in each frame). Papillary muscles and ventricular trabeculations were included with the blood pool. LVM was measured at all frames in the cine acquisition by subtracting the epicardial volume from the endocardial volume, and multiplying by an assumed myocardial density of 1.05 g/ml as above.

**Figure 1:**
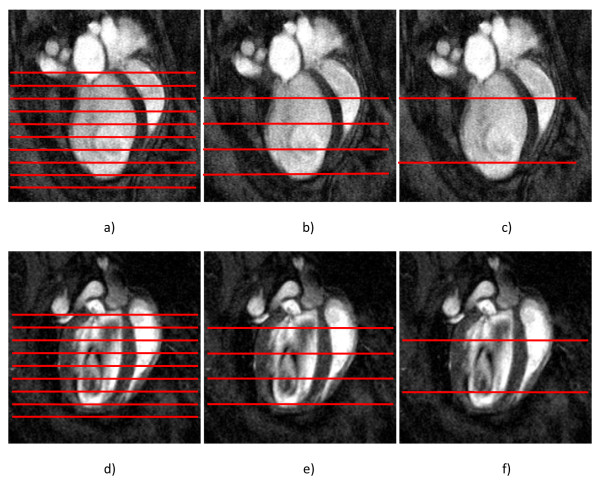
**Location of short axis CMR slices (red lines) included in the a) full b) six slice and c) four slice guide-point modeling analyses of an infarcted mouse, and d) full e) six slice and f) four slice guide-point modeling analyses of a control mouse, overlaid on the long axis four chamber slice (TE/TR = 1.8/4.6 ms; field-of-view (FOV) = 25.6 × 25.6 mm, image size 256 × 256, NAE = 2, bandwidth = 147 kHz)**. Orthogonal two chamber slice not shown.

The GPM analysis was applied to up to three separate studies per mouse: a) a full slice protocol incorporating all SAX and both LAX slices; b) a six slice protocol incorporating four SAX and two LAX slices; and c) a four slice protocol incorporating two SAX and two LAX slices (Figure 1). All analyses were performed blinded to other results and to the ground truth analysis. The six slice protocol was performed by omitting every second SAX slice within the LV, as well as SAX slices at the extreme apical and basal ends of the LV, leaving four SAX and two LAX slices. The four slice protocol was performed by omitting all SAX slices except for two, one in the middle of the basal half and one in the middle of the apical half of the LV, as well as the two LAX slices (Figure 1).

### Statistical Analysis

Agreement in LV mass, EDV, ESV, and EF between standard analysis and GPM were graphically assessed using Bland-Altman analysis. Repeated measures ANOVA was used to compare all GPM results against the standard result. Bonferroni correction was used to perform the post-hoc tests comparing GPM results between protocols.

## Results

**Figure 2:**
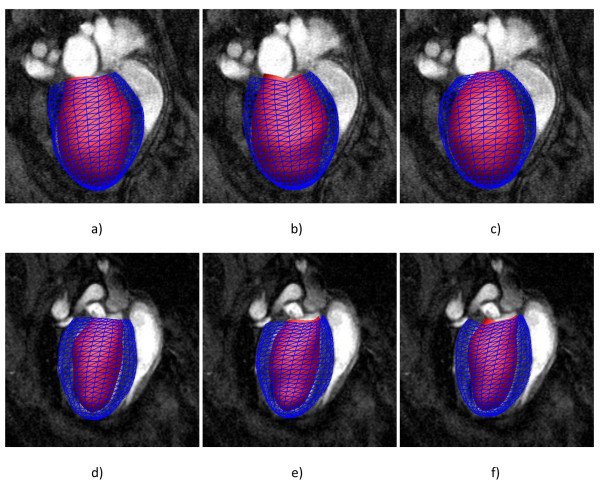
**Three-dimensional model of the LV geometry resulting from a) full b) six slice and c) four slice guide-point modeling analyses of an infarcted mouse, and d) full e) six slice and f) four slice guide-point modeling analyses of a control mouse**. Shaded surface shows the endocardial surface, blue mesh lines show epicardial surface.

Figure 2 shows three-dimensional models of the end-diastolic LV geometry obtained in infarcted hearts from full slice (Figure 2a) and six slice (Figure 2b) protocols, and in normal hearts from the four slice (Figure 2c) guide point modeling protocols, respectively. The shaded surface shows the endocardial surface, while the blue mesh lines depict the epicardial surface. Online additional files include four movies showing full and six slice analyses in an infarcted heart, and full and four analyses in a control heart (Additional file 1 Additional file 2 Additional file 3 Additional file 4). Despite the reduced number of slices used in the modeling process, the ventricular geometry can still be accurately derived. This observation is quantitatively confirmed by the cardiac-functional parameters, which are listed in Table 1 for control mice, and in Table 2 for the infarcted mice (infarct size: 39.1 ± 5.1% - mean ± SD; range: 35 - 48%). While LV mass of control hearts were nearly identical for all analyses methods, LV volumes were slightly higher for GPM than for manual segmentation, resulting in smaller EF values. However, these differences were not statistically significant.

**Table 1:** Cardiac functional parameters for each method, control mice n = 6.

	*Std*	*GPM* *Full*	*GPM* *Six Slice*	*GPM* *Four Slice*	*Std-GPM* *Full*	*Std-GPM Six Slice*	*Std-GPM Four Slice*
LV mass [mg]	105 ± 14	106 ± 10	104 ± 12	105 ± 7	0.4 ± 5.8	-2.0 ± 7.5	-0.6 ± 5.0
EDV [μl]	64 ± 10	68 ± 10	67 ± 9	67 ± 10	-1.2 ± 6.5	-2.0 ± 7.0	-1.4 ± 9.3
ESV [μl]	24 ± 6	28 ± 5	30 ± 6	29 ± 6	1.6 ± 3.7	3.5 ± 4.7	2.2 ± 5.5
SV [μl]	40 ± 6	39 ± 6	37 ± 6	39 ± 6	-2.8 ± 4.3	-5.4 ± 4.9	-3.6 ± 5.4
EF [%]	63 ± 6	58 ± 4	55 ± 6	57 ± 6	-3.2 ± 3.9	-6.4 ± 5.5	-4.0 ± 4.7

Std: standard analysis; GPM: guide point modelling.

**Table 2:** Cardiac functional parameters for each method, infarcted mice n = 5.

	*Std*	*GPM Full*	*GPM* *Six Slice*	*GPM* *Four Slice*	*Std-GPM* *Full*	*Std-GPM* *Six Slice*	*Std-GPM* *Four Slice*
LV mass [mg]	113 ± 10	116 ± 11	117 ± 11	135 ± 18	2.8 ± 4.3	3.4 ± 2.3	22 ± 10*
EDV [μl]	162 ± 20	158 ± 22	160 ± 23	162 ± 22	-3.6 ± 6.8	-2.0 ± 5.7	-0.3 ± 7.1
ESV [μl]	131 ± 20	129 ± 23	131 ± 21	135 ± 22	-1.1 ± 2.5	0.4 ± 4.2	4.5 ± 4.8^+^
SV [μl]	31 ± 6	29 ± 5	29 ± 5	27 ± 3	-2.5 ± 5.4	-1.3 ± 2.1	-4.8 ± 2.8
EF [%]	20 ± 4	19 ± 4	18 ± 3	17 ± 3	-1.1 ± 2.5	-2.4 ± 3.7	-2.9 ± 1.3*

Std: standard analysis; GPM: guide point modelling. *: p < 0.05 manual vs four slice protocol;

^+^: p < 0.05 six vs four slice protocol.

**Figure 3:**
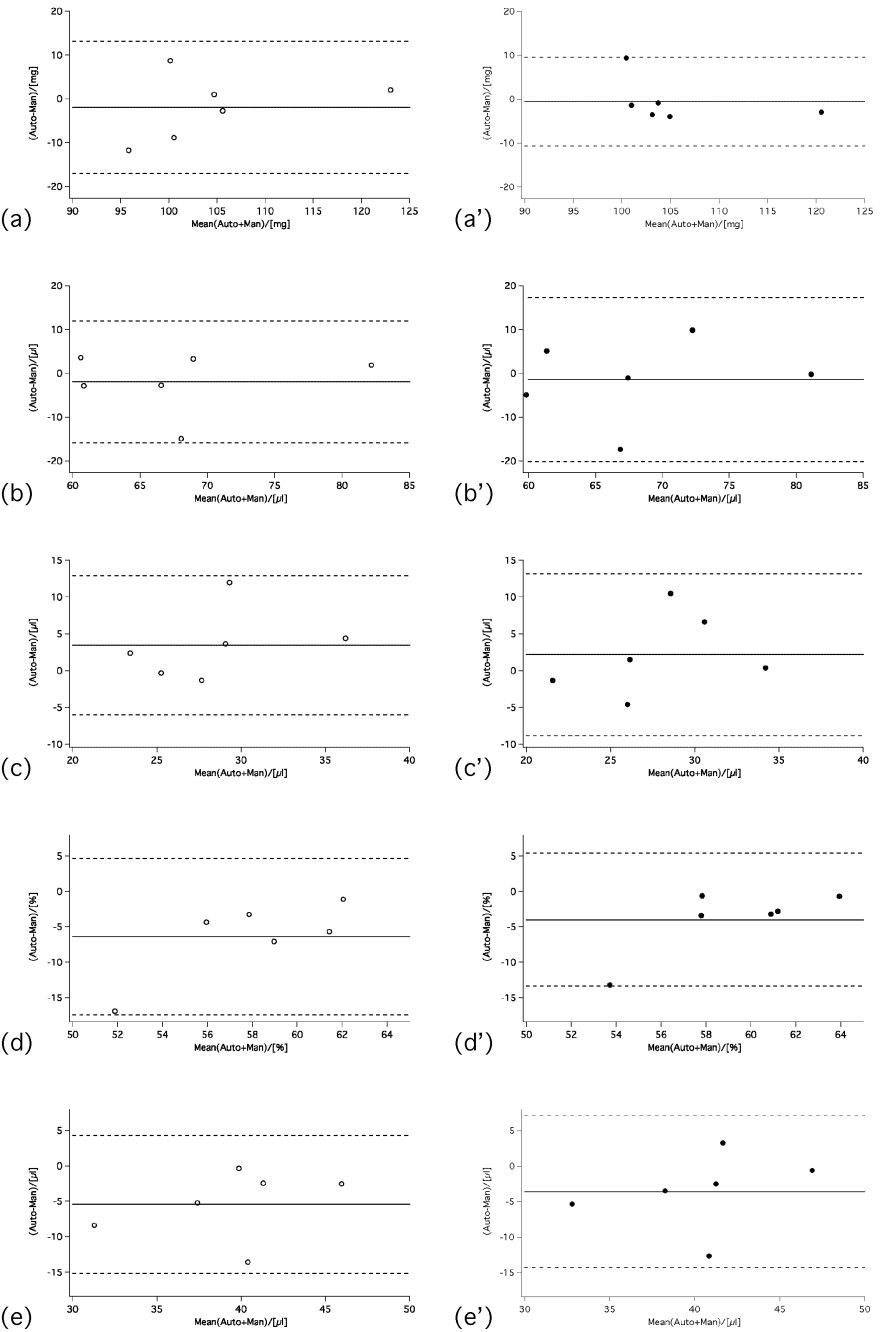
**Bland-Altman plots comparing 3D GPM six slice (open symbols - left column)/four slice (closed symbols - right column, primed panels) protocol versus standard analysis for (a, a') LV mass, (b, b') EDV, (c, c') ESV, (d, d') EF and (e, e') SV obtained in normal mice (*n *= 6)**. The solid lines indicate the bias and the dashed lines the confidence interval (i.e. ± 2SD), respectively.

**Figure 4:**
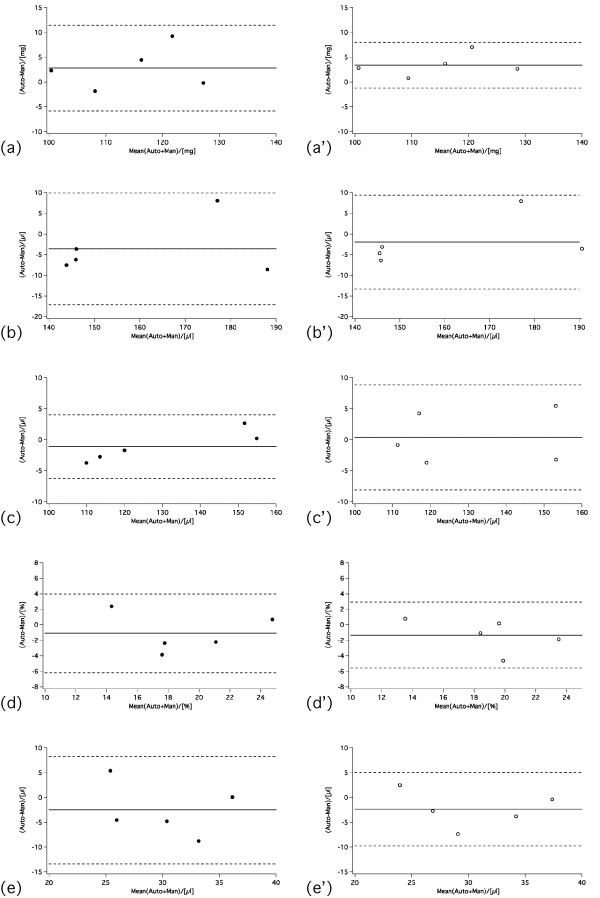
**Bland-Altman plots comparing 3D GPM full slice (closed symbols - left column)/six slice (open symbols - right column, primed panels) protocol versus standard analysis for (a, a') LV mass, (b, b') EDV, (c, c') ESV, (d, d') EF, and (e, e') SV obtained in mice with chronic infarct (*n *= 5)**. The solid lines indicate the bias and the dashed lines the confidence interval (i.e. ± 2SD), respectively.

Bland-Altman plots for (a) LV Mass, (b) EDV, (c) ESV, (d) EF and (SV) are shown in Figure 3 and 4. In infarcted mice, ventricular volumes and LV mass agreed well between standard and GPM full and six slice protocols, and none of the parameters was statistically significant different. Only for the four slice protocol was a significant difference found for LV mass (manual vs four; p = 0.045), ESV (six vs four; p = 0.024) and EF (manual vs four; p = 0.041), respectively. For the control mice, there were no significant differences between standard and GPM full, six or four slice protocols.

## Discussion

A reduction of scanning time in the evaluation of LV mass and volume in murine studies of myocardial infarction is highly desirable, particularly when total examination time is prolonged by multiple measurements under changing conditions, for example before and after stress or loading. The time under anesthesia can be critical for the survival of animals with severe heart failure and reduction of imaging time leads directly to improved outcomes. Although standard manual analysis of contiguous SAX slices has been previously shown to provide accurate estimates of LV mass, compared with autopsy measurements [1], this requires a lengthy examination time given current technology. In normal healthy mouse hearts, 3D GPM analysis of contiguous SAX slices combined with LAX slices has been shown to provide estimates of cardiac function indistinguishable from the standard analysis, with similar scan-rescan reproducibility and in less than a fifth of the analysis time [6]. However, 3D GPM also enables the possibility of reducing the number of slices, since it is a geometric modeling technique, which does not require contiguous SAX slices. In human patients with myocardial infarction, a GPM six slice acquisition protocol including four SAX and two LAX slices has been shown to provide accurate and precise measures of LV mass and volume [7]. This study was therefore designed to test the hypothesis that fewer slices may be required in infarcted mice than are normally obtained, exploiting the ability of the method to interpolate correlated information between slices.

In the current study, we have shown that there was no difference between full and six slice protocols for any of the cardiac functional parameters/LV mass in mice with myocardial infarction involving a substantial part of the left ventricle. Since a typical standard protocol consists of ~10 contiguous SAX slices, this represents a considerable saving in imaging time of ~40% (equivalent to ~10 min with the given experimental parameters). Note that both the full and the reduced slice analyses were performed independently from scratch, equivalent in the real situation to only having the reduced number of slices available.

In control mice without regional dysfunction, further reduction to a four slice GPM protocol resulted in no significant difference in estimated LV mass and volumes. This is perhaps unsurprising since simple one-dimensional formulae have been commonly used clinically for echocardiographic analysis in symmetric left ventricles [10]. CMR evaluation of LV mass and volumes is known to be superior to 2D echocardiography in patients with heart failure or left ventricular hypertrophy due to its ability to construct a 3D representation of the ventricular shape [11]. Although a four slice protocol may be appropriate for a symmetrical ventricle, a moderately larger error is expected in mice with chronic myocardial infarction, due to regionally heterogeneous LV. Note that in this model of myocardial infarction, the errors in volume were acceptable for most purposes, despite being statistically significant.

Our study shows that it is possible to use 3D modelling to obtain accurate mass and volume estimates with a six slice protocol in mice with myocardial infarction. For the first time we provide empirical data for how sparse data can be utilized in mouse studies without trading in accuracy. These empirical data will be useful in future studies of heart disease in the mouse model. Global function parameters can be usefully obtained in mice with relatively large infarcts, and therefore substantial remodelling, using a six slice protocol, and in control mice with a four slice protocol. This raises the possibility of employing the four slice protocol in mice with diffuse disease, such as hypertensive LV hypertrophy, and this should be tested in future studies.

One limitation of the current study is that inter-study and inter-observer reproducibility were not directly evaluated. Rather, different observers trained for several years in the respective techniques performed the GPM and standard analyses independently. It is well established that training is required in order to achieve reproducible results in CMR [12]. A reduced slice acquisition may lead to decreased reproducibility due to the large left-ventricular volumes and thin walls typically observed in mice with chronic myocardial infarction. Therefore, care must be taken in the placement of the contours in order not to introduce an operator bias. A second limitation is that infarct size cannot be derived from the GPM analysis at present and was performed in the manual analysis as described previously [4]. However, late gadolinium enhanced techniques have been also established in murine CMR [13,14] and these may be used in the future with GPM [15] at least in the acute model.

## Conclusions

Reduced acquisition protocols, requiring only two SAX and two LAX slices with globally homogeneous function (such as many genetically modified mice under baseline conditions), and four SAX and two LAX slices in studies of myocardial infarction with regionally heterogeneous function, may be used to provide accurate estimates of LV function in mice.

## Competing interests

AAY is a consultant for Siemens. However, this study was not part of the Siemens consulting contract, and Siemens did not fund any part of this study.

## Authors' contributions

AAY carried out the modeling analysis, participated in the design of the study, and drafted the manuscript. DM carried out the experiments and performed the ground truth segmentation. CL participated in the design of the study, drafting of the manuscript and interpretation of results. SN conceived the study, and participated in its design and coordination and helped to draft the manuscript. JS collated the results, designed and coordinated the study, performed the experiments and helped to draft the manuscript. All authors read and approved the final manuscript.

## Supplementary Material

Additional file 1 **Movie showing control mouse with four slice GPM analysis**. Click here for file

Additional file 2 **Movie showing control mouse with full slice GPM analysis**. Click here for file

Additional file 3 **Movie showing infarct mouse with full slice GPM analysis**. Click here for file

Additional file 4 **Movie showing infarct mouse with six slice GPM analysis**. Click here for file
